# Straightforward Immobilization of Phosphonic Acids and Phosphoric Acid Esters on Mesoporous Silica and Their Application in an Asymmetric Aldol Reaction

**DOI:** 10.3390/nano9020249

**Published:** 2019-02-12

**Authors:** Christian Weinberger, Tatjana Heckel, Patrick Schnippering, Markus Schmitz, Anpeng Guo, Waldemar Keil, Heinrich C. Marsmann, Claudia Schmidt, Michael Tiemann, René Wilhelm

**Affiliations:** Department of Chemistry, Paderborn University, Warburger Str. 100, D-33098 Paderborn, Germany; christian.weinberger@upb.de (C.W.); tatjana.heckel@fh-bielefeld.de (T.H.); schnippe@mail.uni-paderborn.de (P.S.); markusschmitz88@googlemail.com (M.S.); anpeng1108@gmail.com (A.G.); keilw@mail.uni-paderborn.de (W.K.); heinrich.marsmann@uni-paderborn.de (H.C.M.)

**Keywords:** mesoporous silica, organocatalysis, host–guest materials, magic-angle spinning NMR (MAS-NMR)

## Abstract

The combined benefits of moisture-stable phosphonic acids and mesoporous silica materials (SBA-15 and MCM-41) as large-surface-area solid supports offer new opportunities for several applications, such as catalysis or drug delivery. We present a comprehensive study of a straightforward synthesis method via direct immobilization of several phosphonic acids and phosphoric acid esters on various mesoporous silicas in a Dean–Stark apparatus with toluene as the solvent. Due to the utilization of azeotropic distillation, there was no need to dry phosphonic acids, phosphoric acid esters, solvents, or silicas prior to synthesis. In addition to modeling phosphonic acids, immobilization of the important biomolecule adenosine monophosphate (AMP) on the porous supports was also investigated. Due to the high surface area of the mesoporous silicas, a possible catalytic application based on immobilization of an organocatalyst for an asymmetric aldol reaction is discussed.

## 1. Introduction

Ordered mesoporous silica (SiO_2_) materials, such as SBA-15 [1] or MCM-41 [2], are frequently used as support matrices for a large variety of organic functional groups [3]. They are synthesized via sol–gel chemistry-based methods, employing self-assembled arrays of amphiphilic species as porogenic structure directors. The pore sizes can by tuned using various synthesis parameters, such as choice of porogen, utilization of swelling agents, or variation of the reaction temperature. The resulting uniform pore widths typically range from 2 nm to 10 nm, with specific surface areas up to ca. 1000 m^2^·g^−1^. The pore walls exhibit free silanol groups (Si–OH) that may serve for functionalization with organic moieties. Functionalized mesoporous materials have high potential in fields such as drug delivery [4,5,6], separation [7,8], sensing [9], nanotechnology [10], or heterogeneous catalysis [11,12,13,14]. In the latter case, the defined pore diameters allow control over the selectivity of a reaction [15]. In addition, pure silica can also be applied as a catalyst without functionalization [16].

Post-synthetic grafting of linker groups onto the surface of (porous) silica is one of the most frequently employed techniques [3]. The most applied linker moieties are trialkoxylsilane compounds that are linked to SiO_2_ under the release of alcohol [17] or chlorosilanes that form a covalent bond with SiO_2_ under the release of HCl [18]. Yet, these linker precursors have some drawbacks, such as instability toward moisture. Phosphonic acids, on the other hand, are moisture-stable and easy to handle. They are currently standard linkers for various oxidic support materials [19,20]. However, for SiO_2_, just a few complicated procedures of creating covalent bonds with the surface were reported, including adsorption of phosphonic acids via hydrogen bonding, followed by annealing at 120–140 °C for several hours [20,21,22,23,24,25]. In one example, phosphorus acid analogs were attached to a commercial silica gel for chromatography; however, the silica gel needed to be dried extensively prior to use [26]. In addition, phosphonic acids ere attached to fumed silica in anhydrous acetonitrile under N_2_ atmosphere [27]. One simple example was reported that involved immobilization of a phosphonate ester via re-esterification; yet, it was not studied in detail whether a covalent bond was formed [28].

**Scheme 1:**
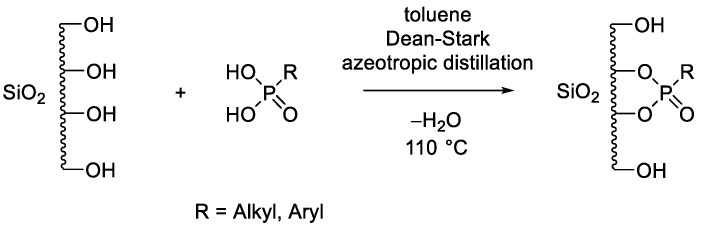
Functionalization of (mesoporous) silica with a phosphonic acid derivative via azeotropic distillation in a Dean–Stark apparatus.

Here, we present a comprehensive study of a straightforward method of immobilizing several phosphonic acids and phosphoric acid esters on various mesoporous silica materials using a Dean–Stark apparatus with toluene as the solvent. This approach can lead to covalently functionalized mesoporous silicas (Scheme 1). A covalent functionalization would have the advantage over adsorption that long-term stability could be reached and that a broader solvent spectrum could be applied with the functionalized material. Since the procedure involves azeotropic distillation, there is no need to dry any of the reactants (phosphonic acids, phosphoric acid esters), the solvents, or the silicas prior to use for the reaction. Our study includes model phosphonic acids, the biomolecule adenosine monophosphate (AMP), and an organocatalyst for an asymmetric aldol reaction.

## 2. Materials and Methods

Unless noted otherwise in this section, all reagents were commercially acquired. Solvents and chemicals were used without further purification. The (2*R*,4*S*)-4-(phosphonooxy)-pyrrolidine-2-carboxylic acid (**5**) was prepared according to a literature procedure [29]. LiChrosorb SI 100 silica gel was purchased from Merck. Mesoporous silicas (SBA-15 [1], MCM-41 [2], and monoliths [30]) were prepared using literature procedures; their specifications are listed in Table 1. Synthesis details and structural characterization (porosity) are shown in the Supplementary Materials. For some reactions, the silicas were pre-dried in a drying cabinet overnight at 100 °C (indicated by “dried”). Since no difference was observed between dried and non-dried silicas, subsequent reactions were performed with the as-obtained material. Loading of the silica was determined by elemental analysis of the carbon content (indicated by *m*_C_) or, where possible, also of the nitrogen content (indicated by *m*_N_).

**Characterization:**^1^H and ^13^C solution-state NMR spectra were recorded on a Bruker AVANCE 500. The deuterated solvents used were deuterium oxide, chloroform, or deuterated methanol. The chemical shifts are given in parts per million (ppm). For solid-state ^29^Si and ^31^P NMR measurements, carried out at 25 or 30 °C; a 300-MHz Tecmag Apollo spectrometer equipped with a wide-bore Oxford magnet was used. The measuring frequencies were 59.563 MHz for ^29^Si and 121.368 MHz for ^31^P. A standard magic-angle spinning (MAS) probe for rotors with a diameter of 4 mm was used. Unless noted otherwise, ^29^Si spectra were obtained at a rotation frequency of 6 kHz, whereas ^31^P spectra were recorded at rotation frequencies of 2 or 8 kHz under proton decoupling. The signal of hydroxy apatite at 2.3 ppm was used as an external chemical shift reference for ^31^P; chemical shifts reported have an error margin of ±0.1 ppm. ^31^P chemical shift tensors were obtained from side-band patterns by means of a Herzfeld–Berger analysis [31] using HBA software [32]. N_2_ physisorption analysis was conducted at 77 K with a Quantachrome NOVA 4000e apparatus. Prior to measurement, samples were degassed at 393 K for 24 h. Pore size evaluation was achieved using the NLDFT-based silica (cylindrical pores) kernel. Fourier-transform infrared (FT-IR) spectra were recorded on a Bruker Vertex 70. Elemental analysis was carried out on a vario MicroCube (elementar) CHNOS analyzer. Powder X-ray diffraction was carried out with a Bruker AXS D8 Advance diffractometer with Cu Kα radiation (40 kV, 40 mA).

**General Functionalization:** Silica (300 mg), phosphonic/phosphoric compound (0.1 equiv.), and absolute toluene (40 mL) were refluxed under N_2_ in a Dean–Stark apparatus for 16 h. Thereafter, the toluene was decanted from the solid. The remaining solid was washed three times with toluene, methanol, and dichloromethane. After this treatment, the solid was dried at 70 °C in a drying cabinet. The level of functionalization and spectral data for each material are presented in the Supplementary Materials.

**2-(Hydroxy(4-nitrophenyl)methyl)cyclohexanone.** (4*R*)-4-Phosphonooxy-l-proline (**5**) functionalized silica (30 mg), *p*-nitrobenzaldehyde (0.20 mmol, 30 mg), and cyclohexanone (9.93 mmol, 0.1 mL) were added to a DMF–water (90−10) volume mixture and stirred for 4 d at 37 °C. The resulting crude product was isolated via column chromatography (eluent: petrol ether–ethyl acetate, 60–40). For silicas, yields, and selectivities, see Table 5.

## 3. Results and Discussion

**Scheme 2:**
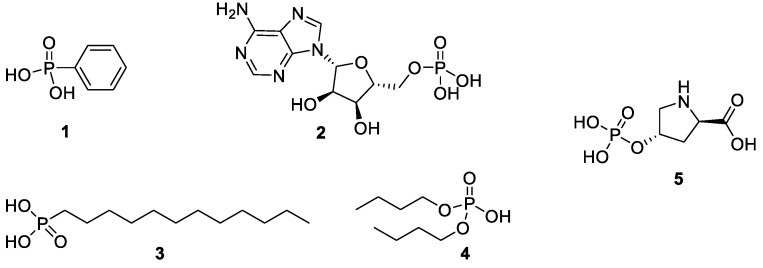
Phosphonic acid and phosphoric acid ester analogs used for functionalization of silica via azeotropic distillation.

To evaluate different phosphonic acid and phosphoric acid ester analogs with alkyl and aryl substituents, phenylphosphonic acid (PPA, **1** in Scheme 2), the biomolecule adenosine monophosphate (AMP, **2**), *n*-dodecylphosphonic acid (DPA, **3**), and di-*n*-butyl phosphate (DBP, **4**) were chosen. Also, (4R)-4-phosphonooxy-l-proline (**5**) [29] was used for a catalytic application.

In addition to the application of different organic moieties, a variety of porous silica materials were also used. Their textural properties are summarized in Table 1.

Before studying phosphonic acids and phosphoric acid esters, preliminary tests were carried out by functionalizing some silica materials with trimethoxysilane as a standard linker via a condensation reaction [17] for comparison. For this purpose, the silicas were dried at 100 °C for 24 h prior to use. The results are shown in Table 2. The data suggest that the pore size has an impact on the loading level. Even though the silica monolith has a smaller Brunauer–Emmett–Teller (BET) surface than SBA-15 silica, a higher loading was achieved. This observation is consistent with the literature [33]. Smaller pores are less accessible for larger molecules. In addition, smaller pores go along with larger specific BET surface areas, which promotes condensation of trimethoxysilane with itself due to surface-adsorbed water and, hence, blocking of the pores, which prevents further functionalization inside the pores. In addition to their pore sizes, the materials may also exhibit differences in the silanol group density (per pore wall area), which might provide further explanation of the different loading levels.

Results for the functionalization with phosphonic acids and phosphoric acids esters are shown in Table 3. This functionalization was achieved using a straightforward method. The various reactants and the respective porous silica materials were stirred in toluene under reflux for 16 h in a Dean–Stark apparatus (Scheme 1), without further pre- or post-synthesis procedures. It was found that pre-drying the silica materials did not result in higher levels of functionalization; the silicas can be applied as prepared in the procedure. The data in Table 3 reveal mostly high levels of loading of the various silica materials with the compounds **1-4** from Scheme 2. The functionalization with PPA (**1**) and DBP (**4**) was similar for all silicas, except for MCM-41. Here, a much lower degree of loading was observed despite the very large BET surface area of MCM-41. This finding may be explained by the fact that MCM-41 exhibited the smallest pores of all silica samples (see Table 1). Obviously, the small pore diameter significantly constrains access of the reactants to the pores as well as their diffusion though the pores. Taking into account that PPA (**1**) reacts very fast with the silanol groups, the PPA (**1**) would react first with the silanol groups at the opening endings of the pores. Thereby, the pore entry becomes too small for further PPA (**1**) to diffuse to the inner part of the pore. For DBA, the effect is smaller due to the fact that DBP (**4**) reacts slower with the silanol groups. The highest level of loading was achieved with AMP (**2**) for all silica materials including MCM-41. This is consistent with the large number of functional groups within this molecule that can form hydrogen bonds. In addition to the AMP molecules chemically bound to the silica surface, additional layers may be formed via H-bonds between adjacent molecules. Lower degrees of loading with DBP (**4**) were observed for all silica materials than with DPA (**3**). This is attributed to the fact that DBP (**4**) exhibits only one hydroxy function to form a bond with the silica surface, which results in a lower binding strength.

**Figure 1:**
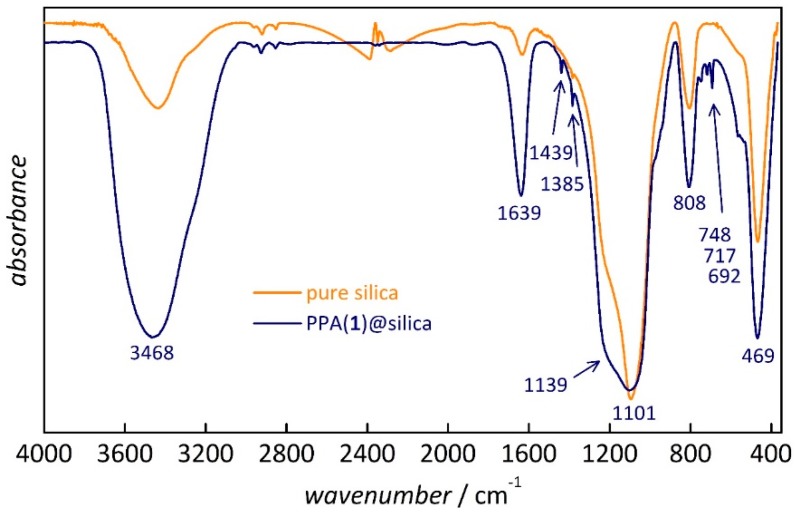
Fourier-transform infrared (FT-IR) spectra of pure silica (monolith) and of the phenylphosphonic acid (PPA) (**1**)-functionalized silica.

The formation of covalent bonds between the silica phases and the respective guest molecules was investigated by FT-IR spectroscopy and by solid-state MAS-NMR spectroscopy. Figure 1 shows example IR spectra of pure silica (monolith) and of the respective PPA(**1**)-functionalized sample. Characteristic absorption bands of amorphous silica appear at 3450 cm^−1^ (s, Si–OH and H_2_O), 1640 cm^−1^ (w, Si–OH and H_2_O), 1180 cm^−1^ (s, Si–O–Si), 800 cm^−1^ (m, Si–OH), and 460 cm^−1^ (m, Si–O–Si) [34]. The functionalized sample exhibits additional signals at ca. 750 cm^−1^, assignable to the P–C stretching vibration, as well as at 1439, 1385, 717, and 692 cm^−1^, assignable to aromatic ring vibrations [21,35,36]. This confirms the presence of the guest species, but not necessarily its covalent bonding to the silica surface. Similar results were obtained for the other samples. ^29^Si MAS-NMR spectra (shown in Supplementary Materials, Figure S4) exhibit weakly resolved Q4 signals with varying fractions of Q3 signals. Due to their low resolution, the ^29^Si NMR spectra did not provide conclusive information on the presence of covalent bonds between the silica surface and the guest molecules. Hence, ^29^Si NMR was not further pursued in this study.

**Figure 2:**
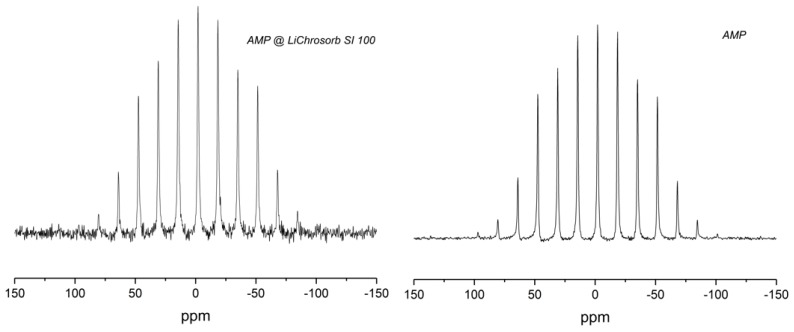
^31^P magic-angle spinning (MAS) NMR-spectrum of LiChrosorb Si 100 loaded with adenosine monophosphate (AMP) (**2**) (left) and of neat AMP (right), measured at a spinning frequency of 2 kHz.

Figure 2 shows an example ^31^P MAS-NMR spectrum of mesoporous silica (monolith) functionalized with the biomolecule AMP (**2**) in comparison with the spectrum of pure AMP. For these samples, as well as the other silicas loaded with AMP (cf. Supplementary Materials, Figure S5), a broad pattern of rotational side bands was observed. The large chemical shift anisotropy indicates that the AMP molecules were immobile. Some of the samples show an additional narrow peak, forming a downfield shoulder of the center band and accounting for at most a few percent of the total spectral intensity. For these peaks, no spinning side bands can be recognized; they may result from a small fraction of mobile AMP molecules or a by-product of the reaction. In order to assign the central peak of the side-band pattern, several measurements at different spinning frequencies were performed; an isotropic chemical shift of −2.0 ppm was determined for all AMP-containing samples. The Herzfeld–Berger analysis [31,32] of the side-band intensities yielded the principal values of the chemical shift tensors, which were 69.2, −1.6, and −73.7 ppm for AMP@LiChrosorb SI 100 and 71.9, −1.8, and −76.1 for neat AMP. The spectral parameters of these two and the other AMP samples are too similar to support the hypothesis of a chemical binding of AMP to the silica surface.

**Figure 3:**
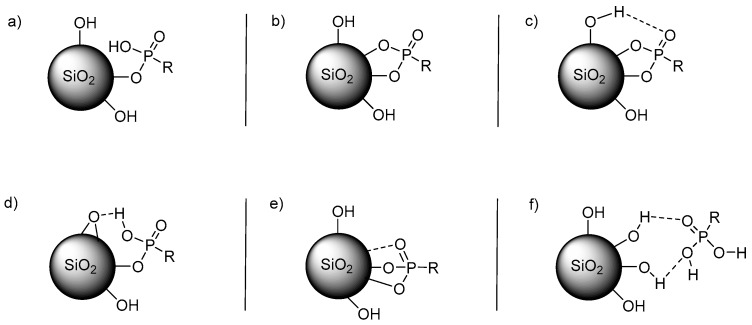
Possible types of attachment of a phosphonic acid to SiO_2_: (**a**) monoester; (**b**) diester; (**c**) diester with hydrogen bonding to a silanol group; (**d**) monoester with hydrogen bonding to a surface oxygen atom; (**e**) diester with Lewis acid/base interaction with a silicon atom; (**f**) pure hydrogen bond interactions.

In a ^31^P MAS-NMR spectrum with PPA (**1**), it would be expected that different signals occur due to the presence of different attached phosphorus atoms to the surface of the silica gel [10]. In Figure 3, six different possibilities are shown, while, in the NMR, only two or three different signals were observable for each sample.

**Figure 4:**
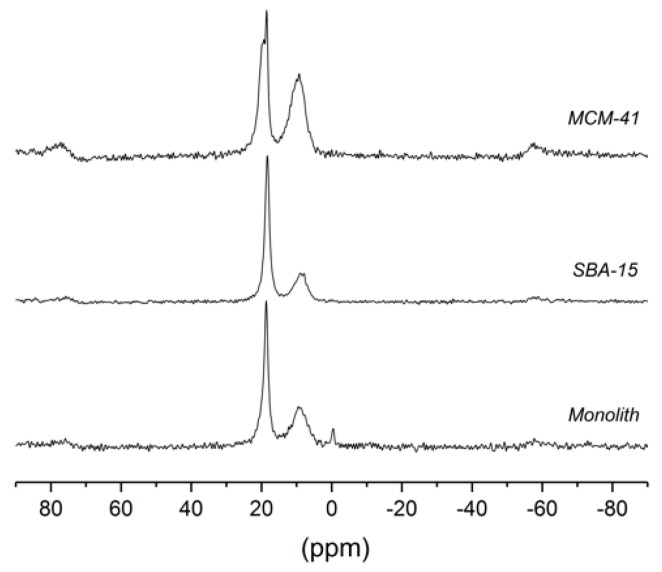
^31^P CP-MAS-NMR spectra of MCM-41, SBA-15, and a monolith functionalized with phenylphosphonic acid (PPA, **1**). The spectra were measured at a spinning frequency of 8 kHz and exhibit spinning sidebands near 80 and −60 ppm.

As can be seen in Figure 4 and Table 4, the ^31^P CP-MAS-NMR spectra of PPA (**1**) attached to different silica gels consisted of four different signals in the range of about 20 to 0 ppm, labeled by increasing numbers from left to right. The peaks outside of this range were spinning side bands. Signals 2 (narrow, between 18.4 and 18.7 ppm) and 3 (broad, between 9.0 and 9.7 ppm) were common to all three samples, but the intensity ratios vary. For PPA@MCM-41, an additional shoulder occurred downfield of 2, whereas PPA in monolithic silica showed a very small additional peak close to 0 ppm. In the literature, the pattern of three peaks was assigned to physisorbed, monodentate and bidentate, and tridentate binding [27].

The width of the narrow peak (2) was reduced significantly under MAS, which means that the width of this peak is partly caused by a residual chemical shift anisotropy and not only by chemical shift variations due to structural disorder. This is consistent with the spectra of other samples (cf. Supplementary Materials, Figure S6) that show spinning side bands of the narrow peak at a spinning frequency of 2 kHz. Thus, the narrow peak may be assigned to partially mobile ligands that cannot reorient isotropically. This could be either a physisorbed species (e.g., via H-bonds) or a monodentate binding; in both cases, the ligand may still be able to rotate about a single bond which reduces the chemical shift anisotropy. The occurrence of two peaks (1 and 2) for MCM-41 may be explained by the occurrence of both physisorption and monodentate binding. The broad peak (3) can be assigned to immobile ligands attached via bi/tridentate binding that makes orientation about a bond axis impossible. The large width of this peak even under MAS indicates structural disorder (different environments).

Silica samples loaded with DPA (**3**) and DBP (**4**) show similar features as PPA (**1**) in their ^31^P NMR spectra (Supplementary Materials, Figure S6). Thus, functionalization of the silica surface by these compounds is confirmed. Only AMP (cf. Figure 2) shows a different behavior. It may also be possible that AMP as the largest guest species considered in this study does not enter the pores at all but simply forms crystals outside of the pore system, which are not removed by the washing sequence (see Section 2). The high "loading" found for AMP (cf. Table 3) would then not entirely correspond to material inside of the pores. 

**Scheme 3:**
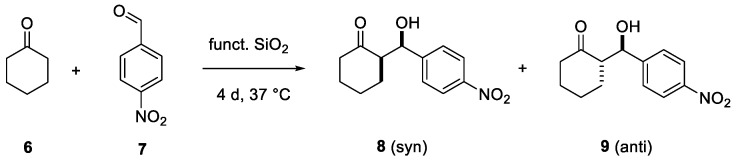
Asymmetric aldol benchmark reaction.

Immobilization of organic moieties in porous silica materials is particularly interesting for heterogeneous organocatalysis. To explore this aspect, (4*R*)-4-phosphonooxy-l-proline (**5**) was immobilized on various porous silica materials in the same way as compounds **1**–**4**. The highest degrees of loading were achieved with LiChrosorb SI 100 (1.234 mmol·g_SiO_2__^−1^) and SBA-15 (1.162 mmol·g_SiO_2__^−1^). These two samples were applied in an asymmetric aldol reaction as shown in Scheme 3; this reaction is catalyzed by l-proline derivatives [37,38]. A heterocatalytic application of **5** in an asymmetric aldol reaction was reported for zirconium oxide as the support material [29]; however, the catalyst could not be recycled due to hydrolysis of the covalent linker bond between **5** and ZrO_2_. It was also shown that a phenylphosphonic acid derivative can be attached permanently to zirconium phosphate [39]. 

**Scheme 4:**
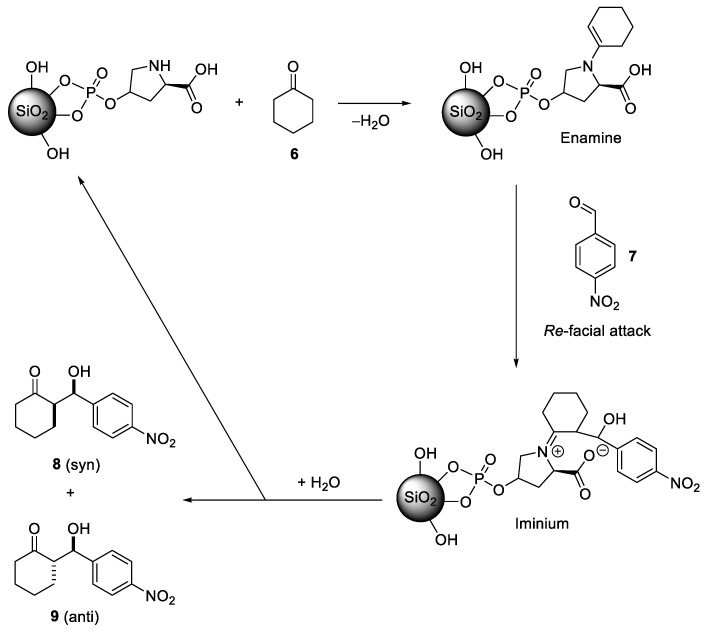
Proposed mechanism of the hetero-catalyzed asymmetric aldol reaction.

For the reaction, 20 mol.% of the functionalized material, 20 equivalents of cyclohexanone and one equivalent of *p*-nitrobenzaldehyde were applied. The spectral data for the obtained two diastereomeres (anti and syn) were consistent with literature values [40,41,42]. The reaction with the pure catalyst (**5**) in solution resulted in a good yield and high selectivities. Slightly lower yields were obtained for the two heterogenic systems of (**5**) immobilized in LiChrosorb SI 100 silica or in SBA-15 silica, respectively (Table 5). The latter resulted in the lowest yield, consistent with its lower degree of loading as compared to the LiChrosorb system. Most remarkably, the immobilization of the organocatalyst does not have any negative effect on the diastereoselectivity (*dr*) or enantioselectivity (*ee*), which is often a problem when chiral catalysts are immobilized. A possible mechanism of the catalyzed reaction is shown in Scheme 4, in analogy to the homogeneous systems [43].

The recycled **5**@SBA-15 material turned out not to be catalytically active. Therefore, we investigated the stability of the functionalized materials from Table 3 against hydrolysis. The materials were exposed to an acidic aqueous solution, five equivalents of a 1 M solution of HCl at room temperature for 24 h. The phosphonic-acid-functionalized materials showed the same level of loading after this treatment, while the materials functionalized with phosphate esters were not stable, as indicated by the absence of carbon in the elemental analysis. Hence, future work will focus on stable organocatalysts linked to the silica substrate via phosphonic acid functions.

## 4. Conclusions

A straightforward method to functionalize various mesoporous silica materials with phosphonic acids and phosphoric acid esters in a Dean–Stark apparatus was presented. The formation of covalent bonds was proven by ^31^P CP-MAS-NMR spectroscopy. These results were exploited to tether a homogeneous organocatalyst via a phosphoric ester group to porous silica, which resulted in good yields and high selectivities in an asymmetric aldol reaction.

## Figures and Tables

**Table 1:** Textural properties of porous silica materials.

Silica	*A*_BET_^[a]^ (m^2^·g^−1^)	*V*^[b]^ (mL·g^−1^)	*d*^[c]^ (nm)
Monolith	206	1.46	30.5
LiChrosorb SI 100	276	1.04	14.0
SBA-15	629	0.75	6.3
MCM-41	1279	0.80	3.7

^[a]^ (Brunauer–Emmett–Teller (BET) specific surface area; ^[b]^ specific pore volume; ^[c]^ average pore width.

**Table 2:** Functionalization of silicas with trimethoxysilane.

Silica	Loading (mmol^·^g_SiO_2__^−1^)
Monolith	0.777
LiChrosorb SI 100	0.562
SBA-15	0.508

**Table 3:** Functionalization of the silicas with different phosphonic and phosphoric analogs. PPA—phenylphosphonic acid; AMP—adenosine monophosphate; DPA—*n*-dodecylphosphonic acid; DBP—di-*n*-butyl phosphate.

Silica	Loading (mmol·g_SiO_2__^−1^)			
	PPA (1)	AMP (2)	DPA (3)	DBP (4)
Monolith	0.527	2.489	-	-
LiChrosorb SI 100^@^	0.742	1.035	0.636	0.470
SBA-15	0.561	0.999	0.568	0.422
MCM-41	0.010	1.049	0.466	0.264

**Table 4:** ^31^P CP magic-angle spinning (MAS) NMR shifts of monolith, SBA-15, and MCM-41, functionalized with phenylphosphonic acid (PPA, **1**).

Silica	Chemical shift/ppm			
	Signal 1	Signal 2	Signal 3	Signal 4
Monolith	-	18.7	9.3	−0.4
SBA−15	-	18.4	9.0	-
MCM 41	19.5	18.5	9.7	-

**Table 5:** Asymmetric aldol reaction carried out by using (4*R*)-4-phosphonooxy-l-proline (**5**) as a catalyst in solution or immobilized in porous silica materials.

Catalyst	Loading (mmol·g_SiO_2__^−1^)	Yield ^[a]^ (%)	*dr*^[b]^ (%)	*ee*^[c]^ (%)	
			anti/syn	anti	syn
Pure **5**	-	62	93/7	96	n.a.
**5**@LiChrosorb	1.234	53	95/5	97	12
**5**@SBA−15	1.162	47	92/8	97	5

^[a]^ isolated yield; ^[b]^ determined by ^1^H NMR; ^[^^c^^]^ determined by chiral HPLC; n.a.—not applicable.

## Supplementary Materials

The following are available online at https://www.mdpi.com/2079-4991/9/2/249/s1: Synthesis procedures for SBA-15, MCM-41, and silica monoliths, as well as their functionalization; Figure S1: physisorption data porous silica materials; Figure S2: low-angle X-ray diffraction (XRD) data of SBA-15 and MCM-41; Figure S3: FT-IR spectra; Figure S4: ^29^Si-MAS-NMR spectra; Figures S5 and S6: ^31^P-MAS-NMR spectra.
