# Supplementary material for: Straightforward Immobilization of Phosphonic Acids and Phosphoric Acid Esters on Mesoporous Silica and Their Application in an Asymmetric Aldol Reaction
Source: Nanomaterials (Basel). 2019 Feb 12;9(2):249. doi: 10.3390/nano9020249 (PMC6409583; doi:10.3390/nano9020249)
Supplement: Supplementary file 1 [file nanomaterials-09-00249-s001.pdf]

Straightforward Immobilization of Phosphonic Acids and Phosphoric Acid Esters on Mesoporous Silica and their Application in an Asymmetric Aldol Reaction

Christian Weinberger, Tatjana Heckel, Patrick Schnippering, Markus Schmitz, Anpeng Guo, Waldemar Keil, Heinrich C. Marsmann, Claudia Schmidt*, Michael Tiemann* and René Wilhelm*

Supplementary Material

Synthesis of SBA-15 silica:

Pluronic P-123 (16.0 g, M_n~5800 g mol^{-1}, Sigma-Aldrich) was dissolved in deionized water (480.0 mL) and hydrochloric acid (48.0 mL, 37 %, Stockmeier). After the addition of TEOS (37.0 mL) the mixture was stirred at 35 °C for 24 h, transferred to a glass-lined autoclave and hydrothermally treated at 80 °C for 24 h. The precipitate was filtered, washed with deionized water and dried at 120 °C prior calcination in a tube furnace at 550 °C for 5 h with a heating ramp of 2.5 °C min^{-1} in air.

Zhao, D.; Feng, J.; Huo, Q.; Melosh, N.; Fredricksen, G.H.; Chmelka, B.F.; Stucky, G.D. Triblock copolymer syntheses of mesoporous silica with periodic 50 to 300 angstrom pores. *Science* **1998**, *279*, 548-552.

Synthesis of MCM-41 silica:

Cetyltrimethylammonium bromide (9.6 g, Sigma-Aldrich) was dissolved in deionized water (480.0 mL) and an ammonia solution (41.0 mL, 25 %) and stirred for 5 min. After the addition of TEOS (40.0 mL) the mixture was stirred at room temperature for 24 h, filtered off, washed with deionized water and dried at 120 °C. Calcination was done in a tube furnace at 550 °C for 5 h with a heating ramp of 2.5 °C min^{-1} in air.

Kumar, D.; Schumacher, K.; du Fresne von Hohenesche, C.; Grün, M.; Unger, K. K. MCM-41, MCM-48 and Related Mesoporous Adsorbents: Their Synthesis and Characterisation. *Colloids Surf., A* **2001**, 109-116.

Synthesis of silica monolith:

Polyethylene glycol (0.616 g, PEG, 35000 g mol^{-1}, Sigma-Aldrich) was dissolved in deionized water (6.0 mL) and nitric acid (0.389 g, 30 %, Stockmeier). After the addition of tetraethyl orthosilicate (5.0 mL, TEOS, Sigma-Aldrich) the mixture was stirred until a clear solution was obtained. The H_2O : HNO_3 : TEOS : PEG molar ratio of the sol was 14.7 : 0.25 : 1.05 : $7.8 \cdot 10^{-4}$. The solution was transferred to a 96-well microplate, covered and aged for 72 h at 40 °C. Afterwards, the monoliths were transferred to a one molar ammonia solution (Stockmeier) at 90 °C for 24 h, followed by washing with deionized water and drying for 48 h between 40-80 °C. Calcination was carried out in a tube furnace at 550 °C for 5 h with a heating ramp of 0.5 °C min^{-1} in air. Before functionalization the monoliths were grinded.

Smått, J.-H.; Schunk, S.; M. Lindén, M. Versatile Double-Templating Synthesis Route to Silica Monoliths Exhibiting a Multimodal Hierarchical Porosity. *Chem. Mater.* **2003**, *15*, 2354-2361.

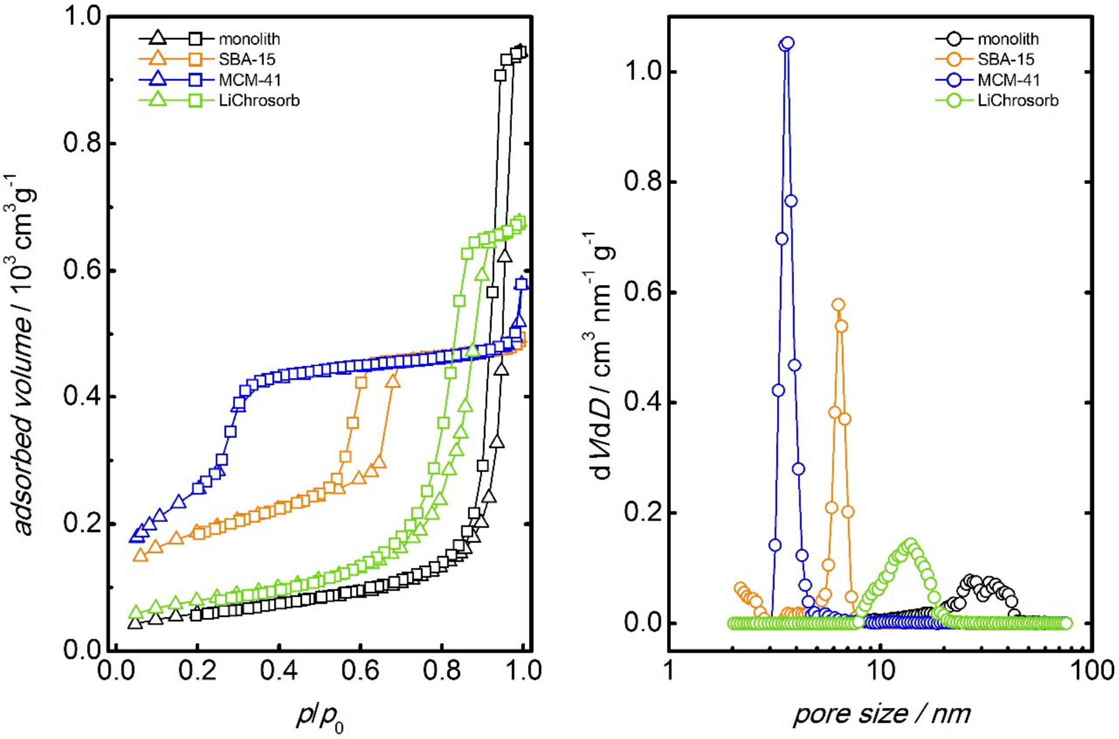

Figure S1. N_2 physisorption isotherms of the monolith, SBA-15, MCM-41 and LiChrosorb.

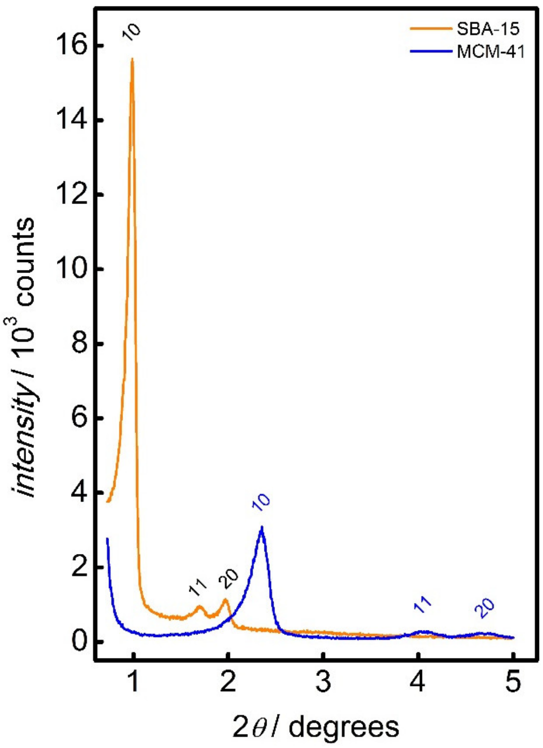

Figure S2. Low angle X-ray diffraction of of SBA-15 and MCM-41.
